# Turning bubbles on and off during boiling using charged surfactants

**DOI:** 10.1038/ncomms9599

**Published:** 2015-10-21

**Authors:** H. Jeremy Cho, Jordan P. Mizerak, Evelyn N. Wang

**Affiliations:** 1 Department of Mechanical Engineering, Massachusetts Institute of Technology, 77 Massachusetts Avenue, Cambridge, Massachusetts 02139, USA

## Abstract

Boiling—a process that has powered industries since the steam age—is governed by bubble formation. State-of-the-art boiling surfaces often increase bubble nucleation via roughness and/or wettability modification to increase performance. However, without active *in situ* control of bubbles, temperature or steam generation cannot be adjusted for a given heat input. Here we report the ability to turn bubbles ‘on and off' independent of heat input during boiling both temporally and spatially via molecular manipulation of the boiling surface. As a result, we can rapidly and reversibly alter heat transfer performance up to an order of magnitude. Our experiments show that this active control is achieved by electrostatically adsorbing and desorbing charged surfactants to alter the wettability of the surface, thereby affecting nucleation. This approach can improve performance and flexibility in existing boiling technologies as well as enable emerging or unprecedented energy applications.

## Abstract (web summary)

Bubble generation during boiling is essential to power generation and heating/cooling systems, but it remains uncontrollable even with state-of-the-art surface engineering. Cho *et al*. electrostatically attract surfactants to the surface, on which bubble nucleation is manipulated in an on-demand manner.

## Graphical abstract

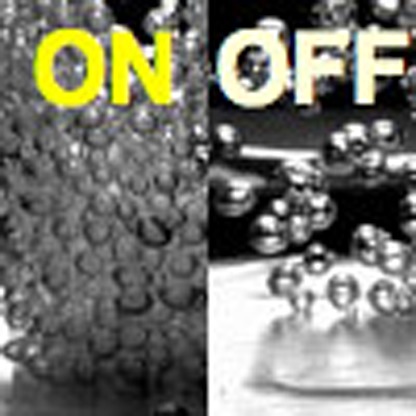

The majority of heating, cooling and electricity generation systems^1^,^2^ rely on boiling to transfer a large amount of heat with a minimal difference in temperature. The efficacy of this process is quantified by the heat transfer coefficient (HTC), which is defined as a ratio of the heat flux, *q*′′, and the temperature difference between the boiling surface and the bulk fluid temperature (superheat), *T*_surf_−*T*_sat_.


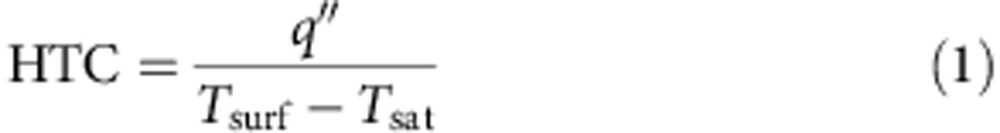


To improve the HTC, wettability is typically decreased to promote vapour generation through increased bubble nucleation^3^,^4^,^5^. This is because a less wettable surface is energetically favourable for nucleation^6^. However, too much nucleation can be detrimental at high *q*′′ when the critical heat flux (CHF) is reached. At CHF, densely packed bubbles coalesce and form a vapour film, which markedly raises the surface temperature, lowers the HTC and can cause device failure. As such, some boiling surfaces are designed to prevent CHF where bubble nucleation is suppressed by increasing wettability^7^. Consequently, increasing wettability alone lowers HTCs in the low *q*′′ range due to more energy being dissipated by convection rather than vapour generation. However, most approaches^8^,^9^ have used micro- and nanostructures to augment the number of nucleation sites to counteract this. Nonetheless, boiling performance is highly dictated by the behaviour of bubbles^10^, which cannot be actively controlled on a particular surface since wettability and surface morphology is typically fixed. Ideally, the HTC would be optimized for a wide range of *q*′′, where bubble nucleation is altered on demand such that more is nucleated at low *q*′′ and less at high *q*′′.

In addition to static surface modifications, surfactants added to the fluid (typically water) in low concentrations increase nucleation^11^,^12^ and the HTC. Enhancement is partly attributed to surfactants adsorbing to the liquid–vapour interface, lowering the liquid–vapour surface tension and allowing bubbles to depart easier^13^. The other contributor to enhancement is adsorption of surfactants to the solid–liquid interface^14^,^15^,^16^ with their hydrophobic components in contact with water to create a hydrophobic (non-wetting) ‘coating' that promotes nucleation^13^,^17^,^18^,^19^. However, the relative importance of adsorption at the solid–liquid interface compared with the liquid–vapour interface has been unclear.

Here we use electric fields and charged surfactants to directly control solid–liquid adsorption^20^, decoupling it from liquid–vapour adsorption effects. Accordingly, this approach allows active control of the contact angle, and hence nucleation density and boiling heat transfer. Compared with another active method of boiling, electrohydrodynamic (EHD) boiling, active surfactant boiling has the benefit of not requiring large electric potentials (∼1 V compared with ∼10^3^ V) and allowing the use of water as opposed to lower latent heat refrigerants^21^,^22^.

## Results

### Turning on and off nucleation

**Figure 1:**
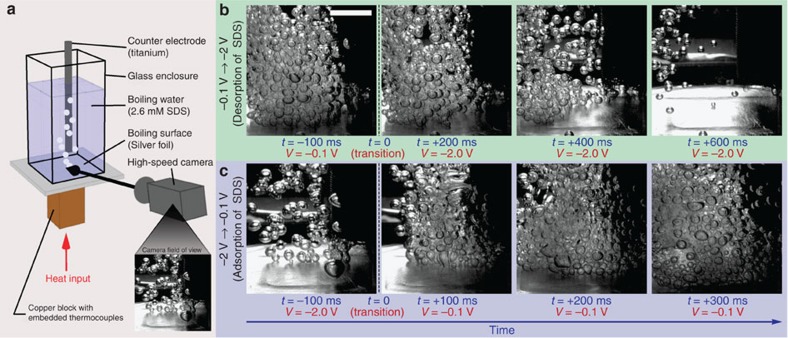
Turning boiling on and off with a potential switch. (**a**) Pool boiling of a solution of 2.6 mM SDS (negatively charged) in DI water at a constant heater power of 60 W with potential applied between the silver foil boiling surface and an immersed titanium counter electrode. A (**b**) −0.1 to −2.0 V switch decreased bubble nucleation within 600 ms due to electrostatic desorption of SDS from boiling surface. A (**c**) −2.0 to −0.1 V switch increased nucleation within 300 ms due to adsorption of SDS (see Supplementary Movie 1). Scale bar, 1 cm.

**Figure 2:**
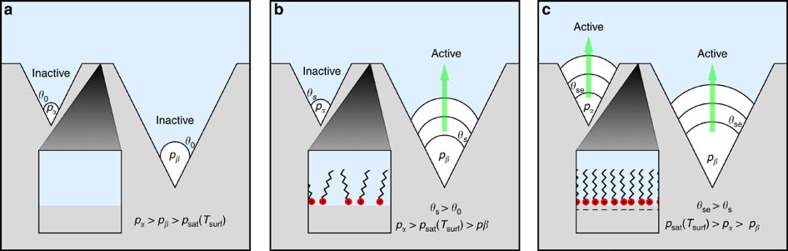
Nucleation behaviour with surfactants and electric potential. A small and large conical cavity at temperature *T*_surf_ are shown under different conditions of surfactant and electric potential. (**a**) When no surfactants are present, the Laplace pressure within the entrapped vapour is high due to a low contact angle. Consequently, the pressure inside both bubbles is too high for saturation conditions; thus, no evaporation occurs. (**b**) When surfactants are added, they adsorb to the solid–liquid interface in a tail-up configuration^19^, which would cause the surface to appear more hydrophobic, increasing the contact angle and lowering the Laplace pressure, which in this case is enough to cause evaporation in the larger cavity. (**c**) When electric potential is applied with surfactants such that they are electrostatically attracted to the surface, the number of surfactants at the solid–liquid interface increases, further increasing contact angle, which is enough to activate both nucleation sites. In all cases, the bubble in the larger cavity starts with a larger initial volume because the volume fraction of the cone occupied by vapour during the trapping (wetting) process is constant since it depends on cone angle and contact angle, which are the same for all cavities^35^.

We demonstrate our ability to actively control bubble nucleation to the extent of turning bubble formation ‘on and off' (Fig. 1; Supplementary Movie 1). This is achieved by electrostatically adsorbing and desorbing charged surfactants using small electric potentials (≤2 V). We applied a constant heat input of 60 W to boil deionized (DI) water with 2.6 mM of negatively charged surfactant sodium dodecyl sulfate (SDS) within a 2 × 2 cm area on a silver boiling surface (Fig. 1a). During boiling, we changed the electric potential applied between the surface and a counter electrode immersed in the fluid (*V*_cell_=*V*_surf_−*V*_counter_) from −0.1 to −2.0 V, which resulted in bubble nucleation immediately subsiding (Fig. 1b). The more negative potential electrostatically repelled the negatively charged SDS away from the surface, diminishing the hydrophobic ‘coating' on the surface, which increased wettability and suppressed nucleation. An illustration of the effect of wettability on nucleation is shown in Fig. 2, with more detailed explanation in Supplementary Note 1 and Supplementary Fig. 1. The weakening of the negative potential from −2.0 to −0.1 V electrostatically attracted SDS to the surface, augmented the hydrophobic ‘coating' and increased nucleation (Fig. 1c). Both nucleation suppression and promotion occurred rapidly (<600 and <300 ms, respectively). The difference in settling times is attributed to nucleation hysteresis^23^. These results show that solid–liquid adsorption/desorption plays a large role in nucleation.

### Square wave potential experiments

**Figure 3:**
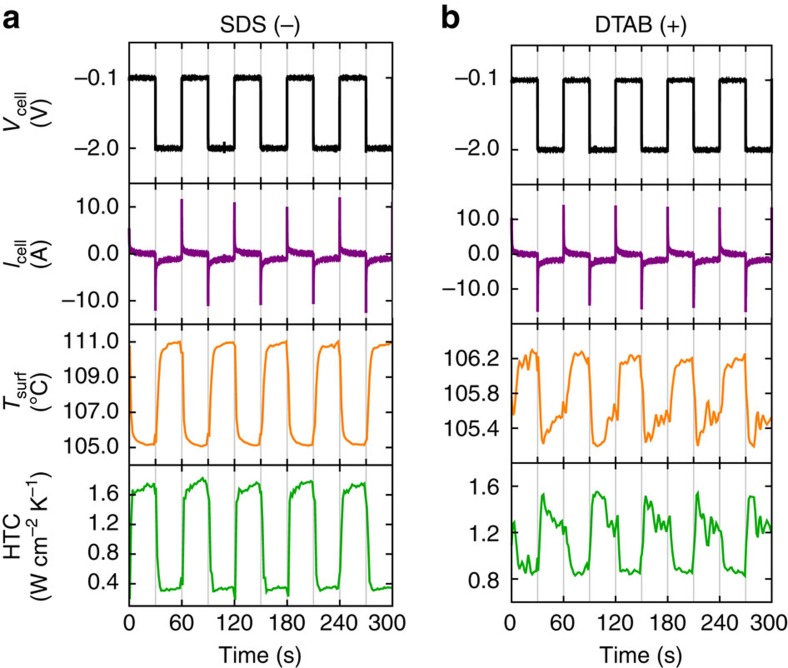
Electrical and thermal responses to a square wave potential. (**a**) Negatively charged SDS had an out-of-phase temperature response and an in-phase HTC response compared with the voltage input. Conversely, (**b**) positively charged DTAB had an in-phase temperature response and an out-of-phase HTC response compared with the voltage input. For both surfactants at 2.6 mM, the input voltage switched between −0.1 and −2.0 V with a period of 60 s and the current response was capacitive.

To investigate the adsorption/desorption mechanism in more detail, we quantified the electrical and thermal response during boiling of solutions of both the negatively charged surfactant SDS and the positively charged surfactant dodecyl trimethylammonium bromide (DTAB) to an applied square wave potential (*V*_cell_ ranged between −0.1 and −2.0 V, 60 s period; Fig. 3). We chose the electrode materials and potential range of −0.1 to −2.0 V based on cyclic voltammetry experiments (Supplementary Fig. 2) to ensure that redox reactions were minimal and that the system behaved like a capacitor for adsorption/desorption behaviour (see Methods and Supplementary Note 2). All surfactants were at a low concentration of 2.6 mM so as not to affect bulk physical properties. This concentration is below the critical micelle concentration of all surfactants in this study (Supplementary Note 3; Supplementary Table 1). The current response for SDS and DTAB (Fig. 3) confirmed capacitive behaviour with a steady-state current not exceeding 60 μA cm^−2^. The thermal response was measured using embedded thermocouples under the boiling surface (see the Methods section) and the results support the adsorption/desorption mechanism. For negatively charged SDS, the temperature increased and the HTC decreased with more negative potential (repelling SDS) since more heat was dissipated through convection as opposed to vapour generation. Accordingly, the temperature was out of phase with potential while the HTC was in-phase with potential (Fig. 3a). In contrast, with positively charged DTAB, the opposite temperature and HTC response was obtained (Fig. 3b) as expected.

### Additional experiments with control additives

Additional experiments where the potential input was a slow triangle wave (quasi-static change in potential) corroborated the square wave experiments (Supplementary Fig. 3). The oppositely charged surfactants SDS and DTAB had opposite thermal responses to the same voltage input. In addition, two negative controls were tested: sodium bromide (NaBr), which is a non-surfactant salt (counterions of SDS and DTAB), as well as *n*-decanoyl-*n*-methylglucamine (MEGA-10), which is a nonionic surfactant. There was no change in nucleation, temperature or HTC with potential for the negative controls (Supplementary Figs 3 and 4). Direct measurements of the advancing contact angles with NaBr, MEGA-10, SDS and DTAB under boiling conditions with different potentials (Supplementary Fig. 5) were consistent with the observed changes in nucleation. In fact, the contact angle of DTAB increased with voltage magnitude as expected; this demonstrates a phenomenon that is completely opposite from electrowetting. Furthermore, the contact angle measurements and triangle wave experiments rule out other mechanisms for actively controlled boiling, that is, EHD or electrolysis, both of which do not rely on charged surfactants (Supplementary Note 4). Moreover, the applied potentials remained in the purely capacitive region (−0.1 to −0.8 V) and were three orders of magnitude smaller than that in EHD boiling^24^. Since the system is capacitive, the number of adsorbed charged surfactants, alteration of boiling nucleation, temperature and HTC (Supplementary Fig. 3) should gradually change with potential as was observed (that is, there is no critical voltage at which these changes suddenly occur). These results indicate that charged surfactants that electrostatically adsorb to the surface decrease wettability and increase nucleation.

### Field-induced tunability of heat transfer performance

**Figure 4:**
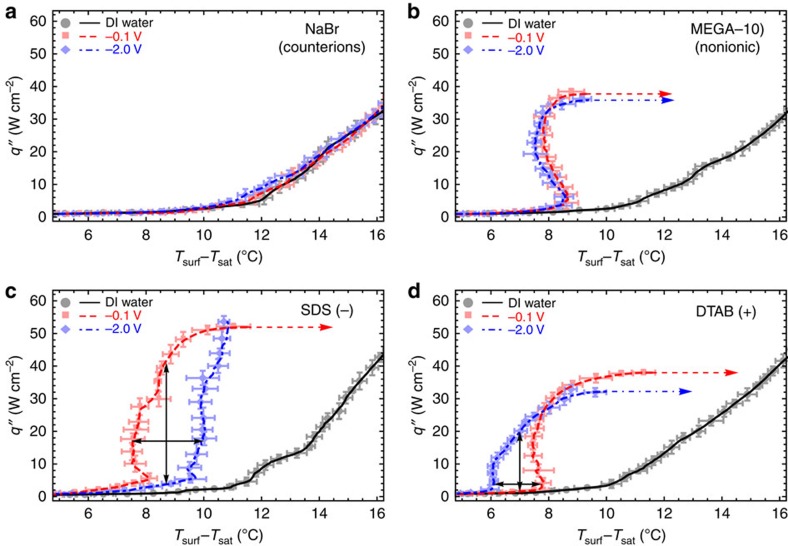
Boiling curves showing tunability of charged surfactants. Plain DI water (black), −0.1 V (red) and −2.0 V (blue) boiling curves for 2.6 mM (**a**) NaBr, (**b**) MEGA-10, (**c**) SDS and (**d**) DTAB. Time averaged data points from an individual boiling experiment with error bars (2 standard deviations in data spread from time averaging) and moving averages (lines) are shown. Boiling was not affected by voltage for (**a**) NaBr and (**b**) MEGA-10. For (**c**) negatively charged SDS, the boiling curve at −2.0 V was right-shifted with higher CHF compared with −0.1 V. For (**d**) positively charged DTAB, the boiling curve at −2.0 V was left-shifted with lower CHF compared with −0.1 V. The maximum change in HTC (tunability) at constant *q*′′ (horizontal arrows) and constant superheat (vertical arrows) are shown for SDS and DTAB.

The performance and degree of field-induced tunability of boiling were quantified by obtaining and analysing boiling curves—the relationship between *q*′′ and superheat (Fig. 4). In these experiments, 2.6 mM of either NaBr, MEGA-10, SDS or DTAB was added to DI water and a *V*_cell_ was applied while the heater power was varied in a quasi-static manner. The boiling curves of NaBr and MEGA-10 at −0.1 and −2.0 V did not change with potential (Fig. 4a,b), confirming the negative control results. Meanwhile, boiling curves at −0.1 and −2.0 V for the ionic surfactants (SDS and DTAB) deviated from each other as expected. The boiling curve for SDS at −2 V was shifted right compared with the baseline −0.1 V curve due to increased wettability and decreased nucleation. The space between the −0.1 and −2.0 V boiling curves represent the ability to actively control (tune) boiling where the HTC for SDS could be increased up to ∼1,000% over its minimum value at −2.0 V for a given superheat. This HTC increase can be seen in the vertical arrows at 8.7 °C superheat in Fig. 4c, where the top arrow points to 41 W cm^−2^ on the −0.1 V curve and the bottom arrow to 3.7 W cm^−2^ on the −2.0 V curve. Furthermore, the temperature could be varied >2 °C; the black horizontal arrows located at 17 W cm^−2^ in Fig. 4c have a low superheat on the −2.0 V curve of 7.5 °C and a high superheat on the −0.1 V curve of 10 °C. The increase in CHF for SDS at more negative potential (not shown but inferred) is consistent with increased wettability due to surfactants leaving the surface. Conversely, applying a more negative −2.0 V to positively charged DTAB decreased wettability to shift the boiling curve left, increase HTC at low q″, but also decrease CHF compared with the baseline −0.1 V (Fig. 4d). The HTC for DTAB could be increased from its value at −0.1 V up to ∼1,100% at a given superheat; the vertical arrows at 7 °C superheat in Fig. 4d have a high heat flux on the −2.0 V curve of 20 W cm^−2^ and a low heat flux on the −0.1 V curve of 1.6 W cm^−2^. The temperature for DTAB could be varied up to nearly 2 °C by voltage change; horizontal arrows at 3.8 W cm^−2^ in Fig. 4d show a high superheat on the −0.1 V curve of 7.8 °C and a low superheat on the −2.0 V curve of 6.1 °C. The ability to shift the boiling curve left and right as well as modify CHF enables modulation and optimization of performance for a variety of conditions. For instance in Fig. 4d, the blue curve (−2.0 V) is more desirable at lower heat fluxes while the red curve (−0.1 V) is more desirable at higher heat fluxes (above the intersection of blue and red curves at 26 W cm^−2^) owing to its higher CHF. This optimization scheme illustrates the opportunity to develop adaptable boiling devices.

### Spatial control of boiling

**Figure 5:**
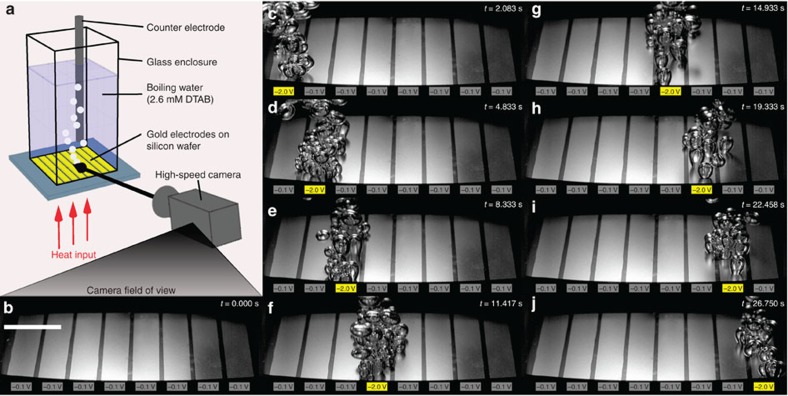
Spatial and temporal control of boiling. A solution of 2.6 mM DTAB (positively charged) in DI water was pool boiled in an experimental set-up (**a**) where eight separate gold electrodes were switched between −2.0 V (yellow) and −0.1 V (grey) to turn on/off bubble nucleation. Images taken at different times with a high-speed camera show (**b**) no nucleation of bubbles when no electrodes were activated, and (**c**–**j**) bubble nucleation only on the particular electrode that was activated. Times in each frame correspond to those in Supplementary Movie 2. Scale bar, 1 cm.

We extended our approach to demonstrate both temporal and spatial control of boiling (Fig. 5) based on our understanding of the mechanism. We fabricated a boiling surface with eight separately addressable gold electrodes insulated from each other, which were heated by platinum resistive heaters underneath (Fig. 5a). With 2.6 mM DTAB, potentials of these electrodes were switched between −0.1 and −2.0 V. We considered −2.0 V the ‘on' state since positively charged DTAB would adsorb to the surface and increase nucleation compared with the −0.1 V ‘off' state. On the backside of each electrode, a relatively uniform heat flux (constant heater power) of ∼1.5 W cm^−2^ was applied across the surface near the onset of bubble nucleation. Figure 5c–j shows our ability to selectively activate bubbles in the area limited to the ‘on' electrode and completely suppress bubbles at the ‘off' electrodes with sub-second precision (Supplementary Movies 2 and 3). Gold electrodes were used due to ease of fabrication. However, we believe the phenomenon to be material independent as long as the potentials are in a capacitive charging regime. Limited testing of copper (Supplementary Fig. 6) has also been shown to produce the field-induced tunability effect.

### Effects of concentration

Our current investigation of the parameter space has shown that concentration plays a large role. For instance, a square wave test with a very low concentration of 0.2 mM DTAB has a less pronounced effect than that of 2.6 mM (Supplementary Fig. 7). This is attributed to the fact that the surface adsorption isotherm of surfactants is monotonic; therefore, surface concentrations would be smaller at lower bulk concentrations^18^,^19^,^25^. In addition, lower concentration solutions are more electrically resistive, which would weaken the electric double layer effect (capacitive charging) due to an ohmic drop in the bulk. This ohmic drop may be quite significant as our testing has shown that tunability is dependent on counter electrode placement (more tunability was observed when the counter electrode was closer). The effect of concentration is also corroborated by superheat measurements of sodium decyl sulfate (S10S) at concentrations from 0 to 27 mM (Supplementary Fig. 8), which show that the tunability effect becomes stronger with higher concentrations. As long as the concentration is below the critical micelle concentration when the boiling curve begins to shift right^11^ (which is the case for all surfactants tested), we expect the field-induced tunability effect to occur.

## Discussion

Our work demonstrates that accurate control of boiling is possible, spatially on the scale of a few millimetres and temporally in the sub-second range using charged surfactants with the application of an electric potential. This study focused on attaining the ability to have adaptable boiling devices; thus, attaining maximum performance in HTC or CHF was not the goal of this study. However, since the link between nucleation and wettability is fundamental and this work involves direct manipulation of wettability, our approach should be able to enhance existing high-performance boiling surfaces with structured^9^ or porous features^7^. Any surfactants adsorbed or desorbed would change the intrinsic contact angle of rough or structured surfaces; thus, the effect could be magnified by Wenzel's model^26^. A thorough parametric study of different materials, geometries, surfactants, voltages and concentrations would likely reveal even more optimal configurations. The pH of solutions is not expected to be a large factor since the zeta potential of metals is approximately several tens of millivolts^27^ and the potentials used here are approximately an order of magnitude higher. Because of this, and the fact that most metals and oxides have similarly low intrinsic contact angles, we do not believe the tunability behaviour is significantly dependent on the choice of material.

A longer term study of overall system robustness would be necessary before electric fields and surfactants can be introduced in real applications. Although in our experiments, no evidence of degradation was observed. No noticeable changes in boiling behaviour were seen during the duration of >10 h of our boiling experiments. Furthermore, no noticeable changes were observed even between experimental runs, which spanned as much as several weeks. Thermal decomposition was likely minimal since surfactants remained well below the thermal decomposition temperatures that are typically in the range of 400 °C (ref. 28). For applications at elevated pressures near the critical point of water (*T*_crit_=374 °C) such as in thermal power plants, surfactants with fluorocarbon tails could be thermally stable up to 600 °C (ref. 29). Electrically, we did not observe any irreversible charging/discharing; thus, we believe the process to be nearly completely reversible, which is consistent with the electrical capacitor model. Furthermore, the use of electrical fields to move ions in industrial applications is not unprecedented as electrodialysis and capacitive deionization techniques are used for desalination purposes^30^. Even if degradation or other irreversible effects were appreciable, we believe that periodic replenishment could be a viable option since only very low concentrations of surfactants are required.

The generality of this approach of surfactant boiling with electric fields suggests that this phenomenon could be utilized in a wide variety of applications. Implementation would be relatively easy since no complicated or micro/nanoscale structures need to be fabricated on the surface. Although our spatial control sample was created using microfabrication techniques, these were only used for millimetre-scale patterning of electrodes on the boiling surface or heaters on the backside and not to build structures that affect nucleation. Spatial control would be beneficial in flow boiling applications where the heat transfer performance and stability can be highly sensitive to the location of bubble nucleation^31^. Overall, temporal control of boiling would be advantageous for non-steady-state applications such as dispatchable power stations, electronics cooling^32^, distributed power^33^ or microfluidic actuators^34^.

## Methods

### Plain surface pool boiling set-up

All pool boiling experiments were performed on a pool boiling rig. For plain surface (silver boiling surface) experiments, 51 μm thick silver foil was roughened with 240 grit (CAMI) sandpaper to provide some nucleation spots and was soldered to the top of a custom-fabricated copper heating block, which could be heated from the bottom by five cartridge heaters (CIR-1029, Chromalox). These cartridge heaters were powered by a programmable high-power supply (KLP-600-4, Kepco). The top section of the copper block had a narrow 2 × 2 cm square cross-sectional area and was insulated to provide one-dimensional (1D) conduction. A glass enclosure (for holding the fluid) was placed on top of the silver foil, sealed by an ethylene propylene diene monomer (EPDM) rubber gasket (Fig. 1a). The top of the glass enclosure was fitted with a custom PEEK covering with port holes for the counter electrode, fluid addition/removal and a coil reflux condenser (QC-6-4, Quarkglass). The fluid side of the condenser was connected to a chiller (RTE-111, Neslab) so chilled water could circulate through the condenser and condense boiled vapour, maintaining a closed loop system. Rope heaters wrapped around the glass enclosure helped maintain saturated conditions. The counter electrode consisted of a 40 × 40 (US) titanium mesh (for high surface area) around a 6.35 mm diameter titanium rod.

To calculate heat flux and measure temperature, four thermocouples spaced 8 mm apart in the constant square cross-section of the copper block just beneath the foil were used. The topmost thermocouple was used for surface temperature measurement. Due to the existence of heat losses (imperfect 1D conduction), the following fin equation was solved and used to fit the thermocouple data to determine the heat flux at the surface.


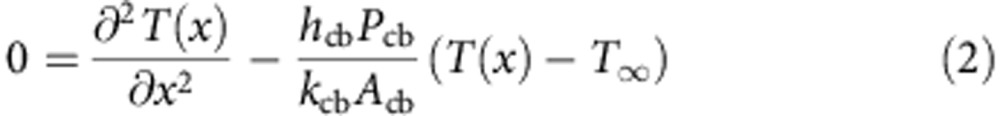


Here, *h*_cb_ is the HTC from the copper block to the ambient, *P*_cb_ is the perimeter of the block, *k*_cb_ is the thermal conductivity of copper, and *A*_cb_ is the cross-section of the block. The fin equation is valid in this case since the Biot number was calculated to be 0.003. The boundary conditions used were 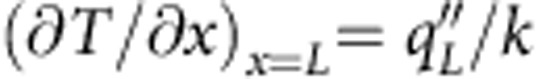 and *T*(*x*=0)=*T*_surf_. An arbitrary value of *L* was applied, while 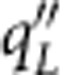 and *h* were used as fitting parameters.

Voltage between the silver foil and titanium counter electrode was applied by a voltage follower op amp and current was measured using a current follower op amp. A digital to analogue converter (DAC) attached to a data acquisition device (U3-HV, Labjack) provided the input signal for voltage and a multimeter (2001, Keithley) measured the voltage across the current follower to obtain current. Since some voltage was lost across the op amp inputs, *V*_cell_ was monitored by a separate multimeter (34401A, Agilent) while a custom LabView program implemented PID control to maintain a desired voltage. An increased voltage loss during the MEGA-10 triangle wave experiments (Supplementary Fig. 3), coupled with DAC range limitations prevented the *V*_cell_ from reaching −0.1 V; thus, −0.2 V was used instead.

Boiling curves were obtained by heating the liquid to a certain power and removing power from the heaters, allowing the surface to cool down from a superheated condition; hence, descending boiling curves were obtained. The cooling rate (∼1 to 2 °C min^−1^) was sufficiently slow since boiling curves obtained using slower cooling rates were nearly identical.

Images and movies were taken using a high-speed camera (Phantom V7, Vision Research) fitted with an single lens reflex lens (EF 28–135 mm f/3.5–5.6 IS USM, Canon) at 640 × 480 resolution and 120 frames per second. A halogen fibre optic light provided illumination.

### Spatially controlled boiling set-up

The spatially controlled boiling surface was created on a silicon wafer: 600–650-μm thick, boron doped, (100) grain orientation and 10–50 Ω cm resistivity (WaferNet). A 1 μm oxide layer (silicon dioxide) was grown thermally through wet oxidation at 1,050 °C. An image reversal photoresist (AZ5214E, AZ Electronics Materials) was spin coated on the polished side after priming the wafer in an HMDS oven at 150 °C. The photoresist was ultraviolet exposed for 1.6 s after soft baking at 95 °C for 30 min. This was then followed by a hard bake on a hot plate at 120 °C for 90 s and flood exposure (ultraviolet radiation) for 60 s. The wafer was then developed using AZ422 MIF developer (AZ Electronics Materials) to obtain the negative image of the thin-film heater. Finally, electron beam evaporation was used to deposit 10 nm titanium for adhesion followed by 100 nm platinum for the heaters. After deposition, the wafer was placed in an acetone bath to remove the photoresist and create the final thin-film heater design. On the unpolished side, an aluminum shadow mask with slots for electrodes 1 cm in width was cut by waterjet and placed on top of the silicon wafer. The wafer was then sputtered with 100 nm of titanium for adhesion and 500 nm of gold electrode material. The unpolished (rough) side was used for boiling to provide some nucleation spots.

Each gold electrode could be switched between −0.1 and −2.0 V. This was achieved by using two positive voltage DAC channels, with one set to 0.1 V and the other set to 2.0 V. Each DAC channel was inverted using a unity-gain inverting op amp to achieve the final negative voltages. For each electrode, voltage from either channel was selected using an SPDT switch, which was controlled by a digital out channel from the data acquisition device. Heaters were controlled using 5 kΩ potentiometers in series. Each heater-potentiometer line was connected in parallel to the high-power supply.

### Contact angle measurements

A 30 × 6 × 13 mm copper block with a 130 μm diameter hole on the underside was fabricated. The underside was polished with a 1 μm polishing paper. The hole was connected to a vapour chamber within the block from which helium flowed through and could exit through the hole. The block was positioned with a slight tilt so that a bubble forming around the hole would eventually slide off due to buoyancy. As the bubble slid off, the advancing contact angle was measured with the high-speed camera and macro lens (MP-E 65 mm f/2.8 × 1–5 Macro Photo, Canon). Contact angles were measured by fitting a circle tangent to the contact line with a custom Mathematica code. The circle was fit using three control points. With automatic variation of control points around the area of interest, the uncertainty was determined. Some main sources of error come from the imperfectly circular profile of the bubbles, ability for software to resolve bubble edges in shadow regions, and camera positioning, all of which may have varied between tests.

### Surfactant preparation

NaBr, MEGA-10, S10S, SDS and DTAB (all from Sigma) were dissolved at a concentration of 173 mM in DI water. Mixtures were sonicated for 1 h at 40 °C for complete dissolution. In most experiments, 6 ml of these solutions were added to 400 ml of DI water using an automatic pipette, bringing the total concentration to 2.6 mM.

## Additional information

**How to cite this article:** Cho, H. J. *et al*. Turning bubbles on and off during boiling using charged surfactants. *Nat. Commun.* 6:8599 doi: 10.1038/ncomms9599 (2015).

## Supplementary Material

Supplementary Information Supplementary Figures 1-8, Supplementary Table 1, Supplementary Notes 1-4 and Supplementary References.

Supplementary Movie 1 Turning boiling on and off with an electric potential. 2.6 mM SDS was boiled on silver foil with a constant 60 W heat input and changing the voltage altered nucleation. The movie is comprised of two parts: (1) real time and 33x slowdown of -0.1 V to -2.0 V transition showing nucleation suppression, and (2) real time and 33x slowdown of -2.0 V to -0.1 V transition showing nucleation promotion.

Supplementary Movie 2 Switching boiling on and off on different individually designated areas. Using a 1-D gold electrode array sample to boil DTAB at 2.6 mM under constant heater power where switching voltages between -2.0 V and -0.1 V turned boiling on and off at individual electrodes. Playback speed is slowed down to 0.25x real time. Times correspond to frames in Fig. 1.

Supplementary Movie 3 Ode to Bubbles. A movie of spatial and temporal activation of nucleation in sync with Beethoven's Symphony No. 9 (Ode to Joy) using a 1-D gold electrode array sample, DTAB at 2.6 mM, constant heater power, and voltages of -2.0 V and -0.1 V. Each electrode corresponds to a musical note on the octave scale. Playback speed is 1.54x real time.
