# Supplementary material for: Turning bubbles on and off during boiling using charged surfactants
Source: Nat Commun. 2015 Oct 21;6:8599. doi: 10.1038/ncomms9599 (PMC4639814; doi:10.1038/ncomms9599)
Supplement: Supplementary Information — Supplementary Figures 1-8, Supplementary Table 1, Supplementary Notes 1-4 and Supplementary References. [file ncomms9599-s1.pdf]

Supplementary Information

Supplementary Figure 1

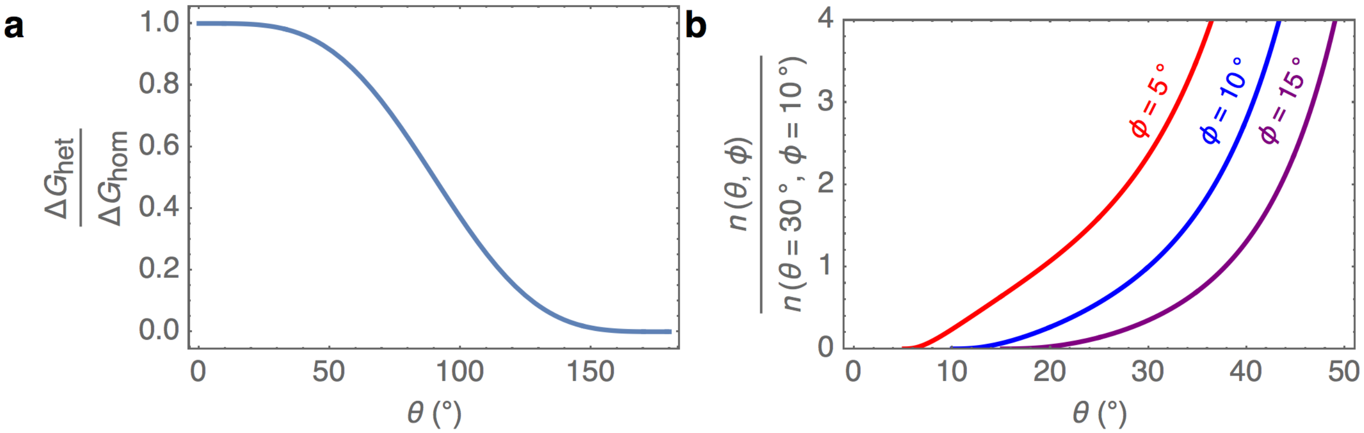

Supplementary Figure 1: **Relationship between wetting and nucleation.** (a) Relative decrease in the energy barrier (free energy change of heterogeneous nucleation, G_{het}, compared to the free energy change of homogeneous nucleation, G_{hom}) to nucleate a bubble as a function of contact angle, θ, according to classical nucleation density (see Supplementary Note 1). (b) Relative nucleation density as a function of contact angle and defect cone angle, ϕ, according to the entrapped vapor theory (see Supplementary Note 1). Nucleation density plotted in comparison to a baseline nucleation density of $\theta = 30°$ and $\phi = 10°$.

Supplementary Figure 2

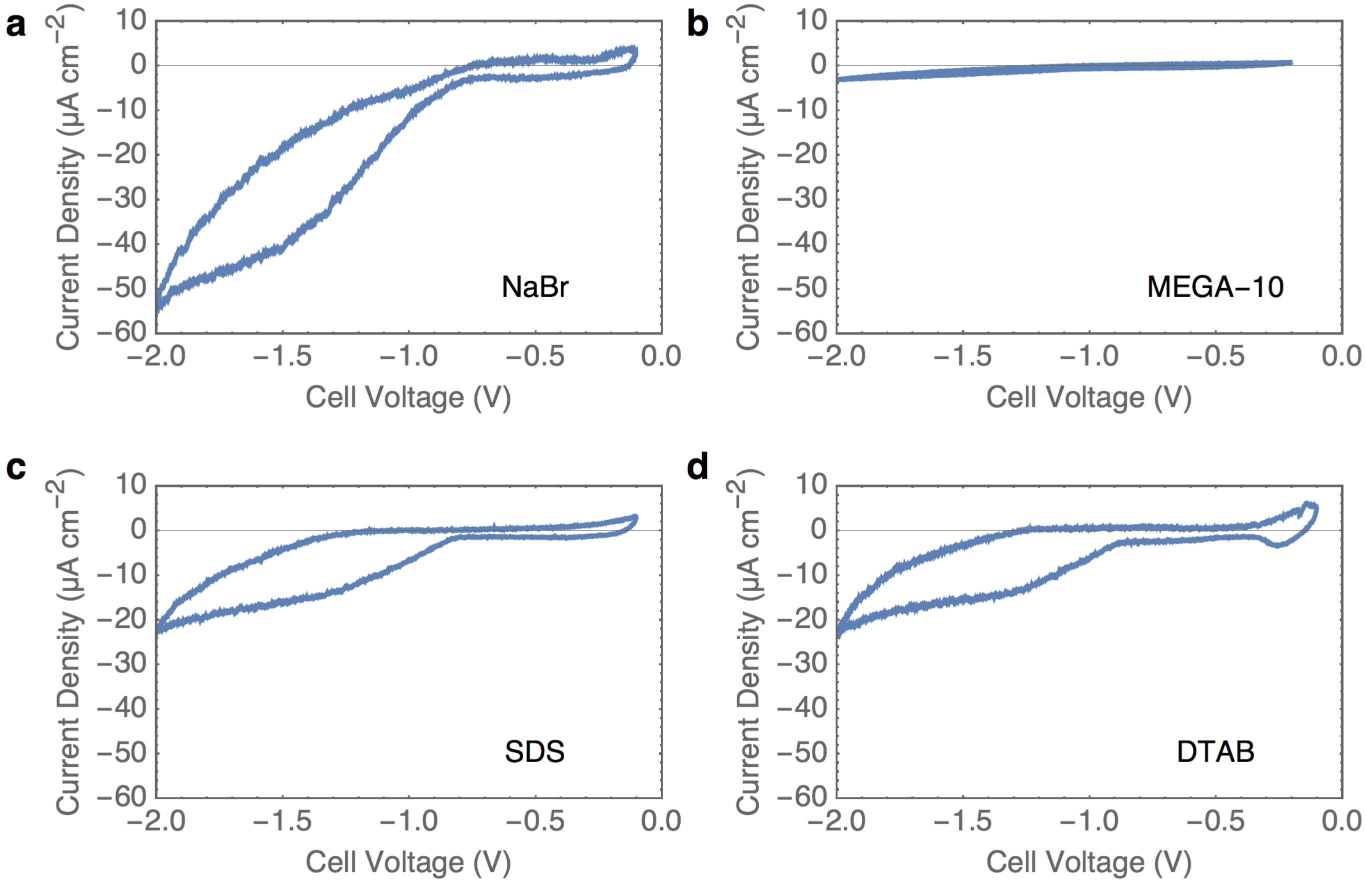

Supplementary Figure 2: **CV scans demonstrate capacitive behavior and low electrolysis.** CV scans were obtained for 2.6 mM (a) NaBr, (b) MEGA-10, (c) SDS, and (d) DTAB at a voltage sweep rate of $10.6\,\mathrm{mV\,s^{-1}}$. At this sweep rate, currents are at their steady-state values (lower sweep rates did not significantly change the current values). From −0.1 V to −0.8 V, all current responses were flat indicating purely capacitive behavior. Beyond −0.8 V, the current response was low ($< 60\,\mu\mathrm{A\,cm^{-2}}$) indicating that electrolysis is negligible (see Methods and Supplementary Note 2).

Supplementary Figure 3

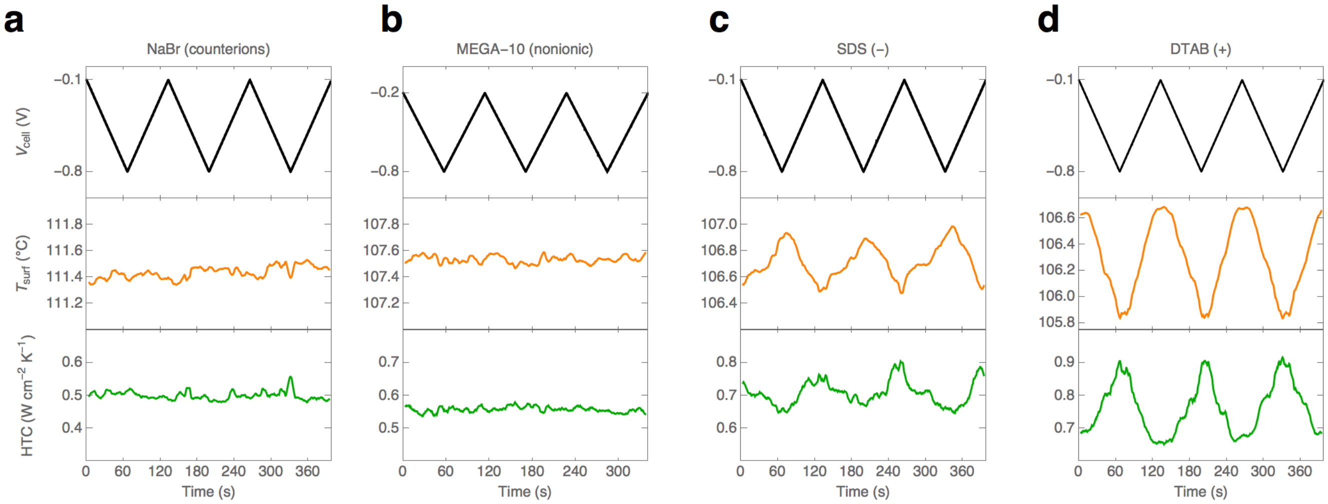

Supplementary Figure 3: **Thermal responses to triangle wave potentials show that only charged surfactants respond and do so according to their charge.** Temperature and HTC for (a) NaBr and (b) MEGA-10 did not respond to the potential input, while (c) SDS and (d) DTAB did respond in opposite directions from each other. The thermal response in SDS and DTAB was continuous within the voltage sweep range. The voltage ranged from −0.1 V to −0.8 V except for MEGA-10 which ranged from −0.2 V to −0.8 V due to limitations of experimental setup (see Methods). Voltage sweep rate was 10.6 mV s^{-1}. This voltage range is in the purely capacitive region according to Supplementary Fig. 2.

Supplementary Figure 4

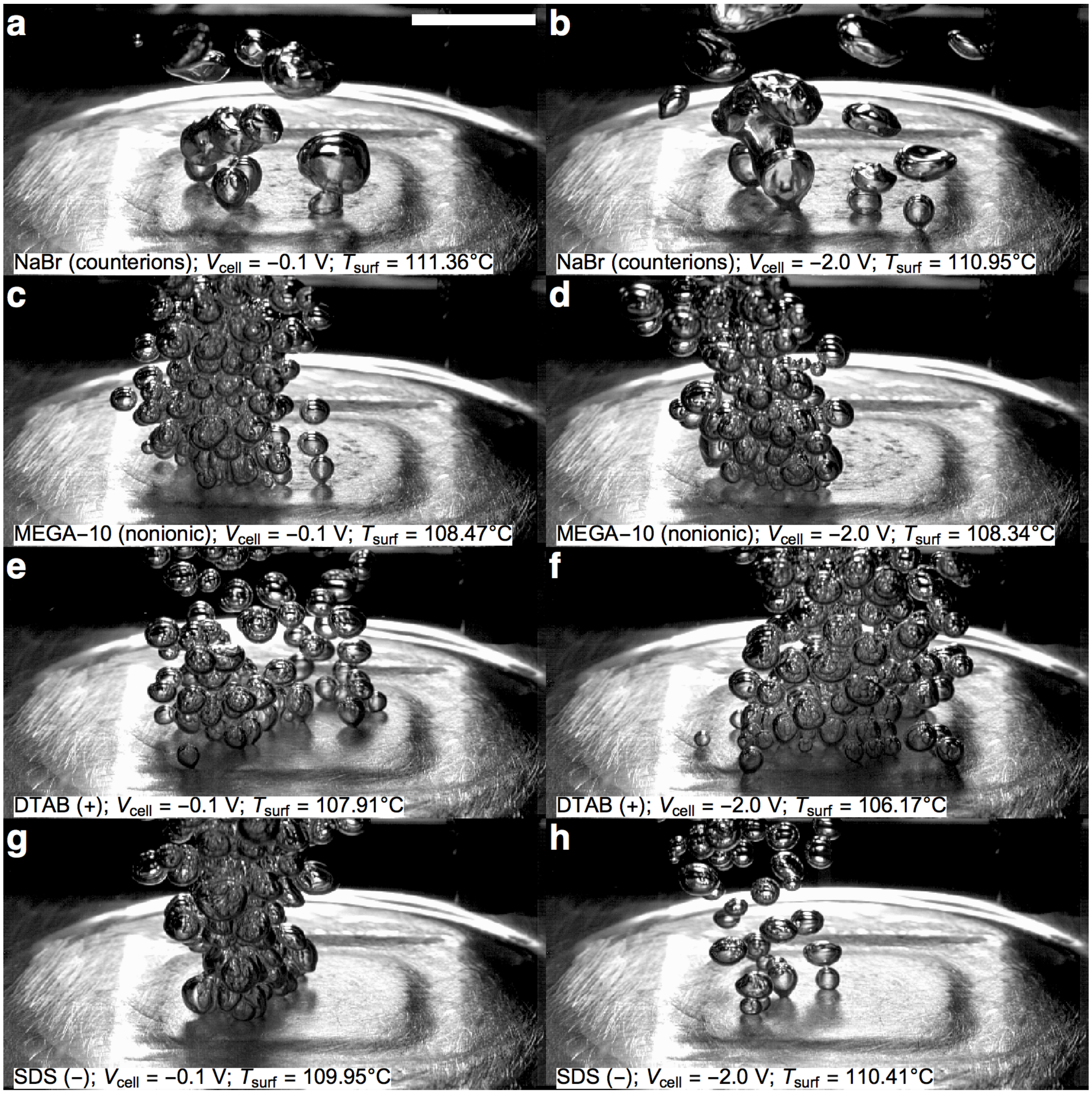

Supplementary Figure 4: **Snapshots during boiling show nucleation changes for charged surfactants**. The heat flux was approximately 5 W cm^{-2}. NaBr at (a) −0.1 V and (b) −2.0 V had no significant change in nucleation density. MEGA-10 at (c) −0.1 V and (d) −2.0 V also had no significant change in nucleation density. For positively charged DTAB, less nucleation and more superheat were observed at (e) −0.1 V than (f) −2.0 V since less heat was being dissipated through phase change. For negatively charged SDS, more nucleation and less superheat were observed at (g) −0.1 V than −2.0 V. The scale bar is 1 cm.

Supplementary Figure 5

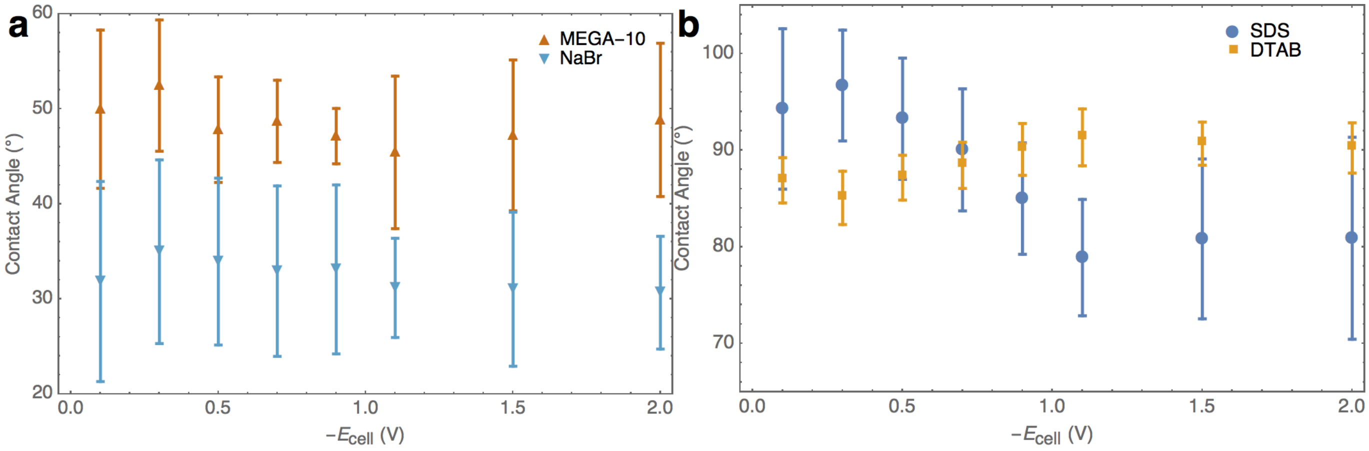

Supplementary Figure 5: **Effect of electric potential on contact angle with controls and charged surfactants**. Advancing contact angle measurements were made at 100 °C. Uncertainty bars represent two standard deviations in contact angle measurement from different image frames and fitting (see Methods). (a) Control additives have little effect on contact angle while (b) charged surfactants show changes in contact angle when the electric potential is changed. Positively charged DTAB is more attracted to the surface at more negative potentials; thus, the increased adsorption results in a higher advancing contact angle. SDS, on the other hand, is more repelled at more negative potentials; thus, the desorption results in a lower advancing contact angle.

Supplementary Figure 6

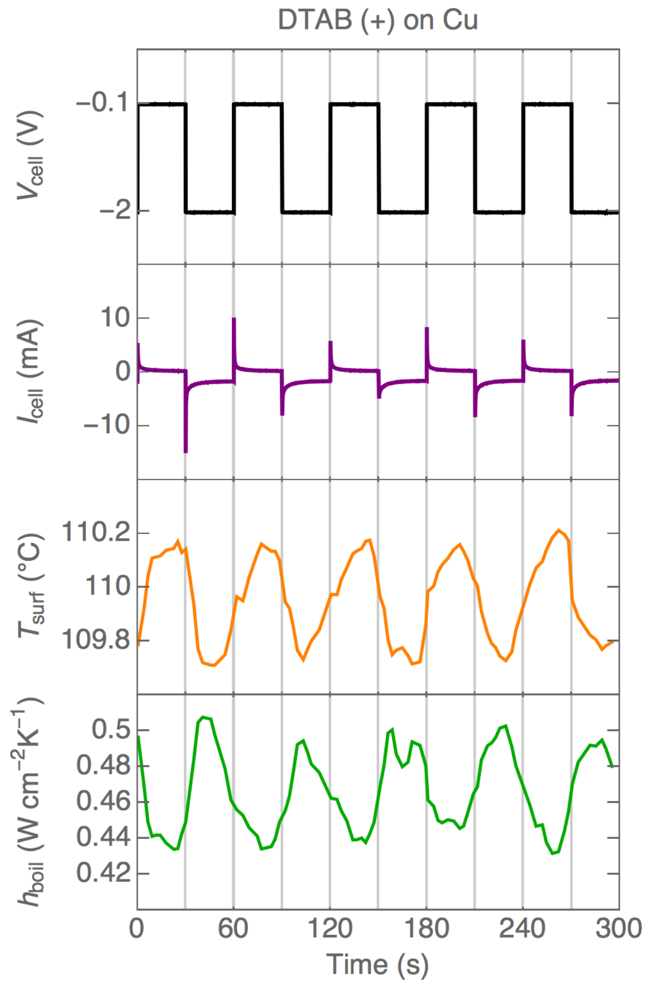

Supplementary Figure 6: **Electrical and thermal response to a square wave potential for DTAB demonstrates tunable boiling effect on copper**. The concentration was 2.6 mM (default concentration used in other experiments). Instead of the silver foil surface (see Methods), a copper foil was used as the boiling surface. This result shows that the tunability effect is mostly independent of the specific material chosen for the boiling surface.

Supplementary Figure 7

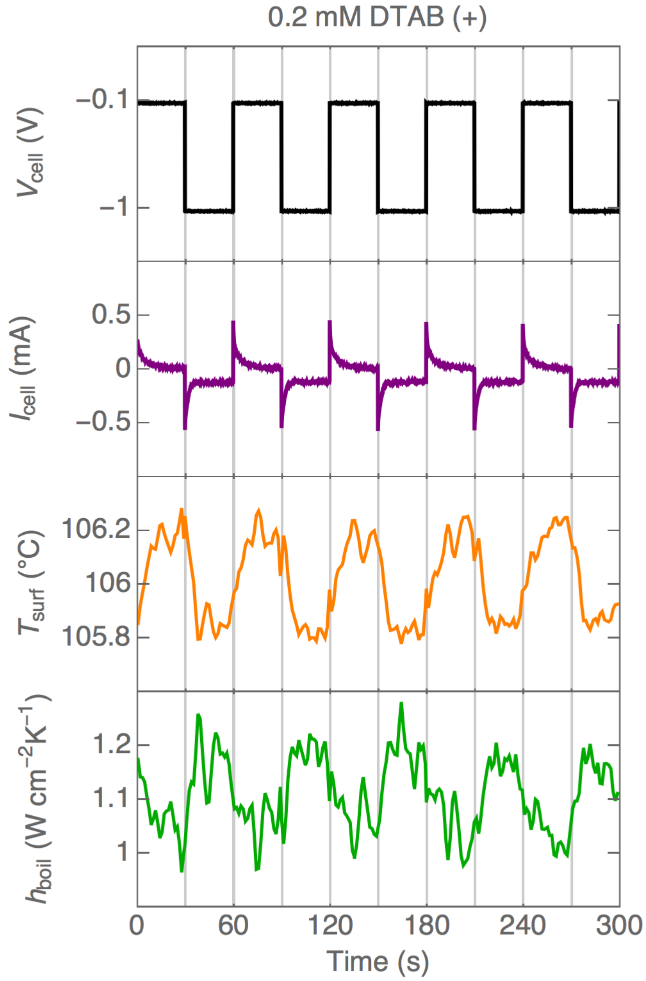

Supplementary Figure 7: **Electrical and thermal response to a square wave potential for DTAB demonstrates tunable boiling effect at a low concentration of 0.2 mM**. This was performed on the default silver boiling surface (same as in the Main text). The response is similar but smaller in magnitude than that of higher concentration DTAB (Fig. 3b). This result demonstrates that tunability decreases at lower concentrations.

Supplementary Figure 8

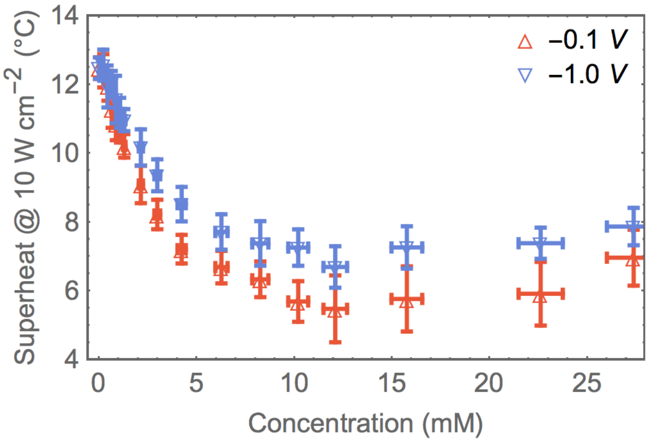

Supplementary Figure 8: **Effect of concentration and electric potential on superheat (at 10 W cm^{-2}) for sodium decyl sulfate (S10S)**. This was performed on the default silver boiling surface (same as in the Main text). The tunability effect (separation between −0.1 V and −2.0 V data points) is lower at low concentrations while plateauing at higher concentrations. This shows that the tunability effect is applicable for a wide range of concentrations as long as the concentration is below the CMC (38.1 mM for S10S). The uncertainty in concentration is $\pm 5\,\%$ while the uncertainty in superheat is two standard deviations in variation due to time-averaging.

Supplementary Table 1

Supplementary Table 1: **Surfactant properties.**

Surfactant	Charge (e)	Tail length (# of carbons)	Molecular weight	CMC at 100 °C (mM)
MEGA-10	0	10	349.46	4.1 ± 1.5
S10S	–1	10	260.33	38.1
SDS	–1	12	288.38	10.4
DTAB	+1	12	308.34	13.9

CMC for MEGA-10 calculated from equations and data given by Sulthana et al. [1]. CMC for S10S, SDS, and DTAB calculated using PREDICT software by Zoeller and Blankschtein [2].

Supplementary Note 1

Nucleation Theory

From classical nucleation theory, the critical energy, ΔG_{het}, required to nucleate on a surface is

$$\Delta G_{\text{het}} = \Delta G_{\text{hom}} \cos^4\left(\frac{\theta}{2}\right)(2 - \cos(\theta)) \tag{1}$$

where ΔG_{hom} is the critical energy required to nucleate homogeneously (fixed for a given supersaturation condition), and θ is the droplet contact angle. From equation (1), increasing the contact angle decreases the energy barrier to nucleate as Supplementary Fig. 1a shows.

The entrapped vapor theory, which is a more complicated but accurate picture of nucleation [3–5], also shows that an increase in contact angle leads to an increase in nucleation. In this model, defects are modeled as conical cavities. For a fluid with a relatively high surface tension such as water, the contact angle is high enough that the liquid is unable to completely wet every defect; thus, vapor/gas is entrapped at these defect sites. According to this theory, nucleation density, n, is a function of contact angle, θ and cone angle, ϕ among other thermodynamic properties and empirically determined parameters.

$$n = \left(\frac{R_{\text{s}}\Omega(\theta,\phi)h_{\text{fg}}\rho_{\text{v}}}{2T_{\text{sat}}\gamma_{\text{lv}}}\right)^m \Delta T^m \tag{2}$$

Here, m is empirically determined to be 6 for most surface/liquid combinations and R_{s} is a parameter that is related to roughness and cavity size and does not change for a surface/liquid combination. γ_{lv} is the dynamic surface tension. The Ω term, which comes from purely geometric arguments, is a complicated expression determined by how a vapor bubble grows out of a conical cavity and can be found in Lorenz' thesis [5]. To illustrate the effect of contact angle and defect geometry, we have plotted in Supplementary Fig. 1b relative nucleation density compared to a baseline nucleation density where $\theta = 30°$ and $\phi = 10°$. From this plot, it is clear that a small increase in contact angle can dramatically increase the nucleation density.

Supplementary Note 2

Voltage and Material Considerations

Many electrochemical considerations were taken into account when choosing materials and the voltage range used in our experiments. The V_{cell} range of -0.1 V to -2.0 V ensures that the working electrode (boiling surface) is being reduced as opposed to being oxidized for silver-titanium and gold-titanium electrochemical systems. Reduction ensures that a pristine metal surface is maintained, which could also be useful in maintaining the quality of boiling surfaces in practice. While experiments were performed at positive voltages with expected results, the boiling surface was visibly oxidized.

Thus, negative voltages were used in this study. Cyclic voltammetry tests show that there is a purely capacitive region near −0.1 V to −0.8 V and electrolysis afterwards as shown in Supplementary Fig. 2. The rate of electrolysis does not exceed 60 µA cm^{-2} (highest for NaBr, lower for surfactants). This corresponds to a power of 2×10^{-4} W cm^{-2}, which is a negligible power consumption compared to the amount of heat transferred. If we assume all of this current contributes to hydrogen production (splitting of water), then the hydrogen generation rate at 100 °C and atmospheric pressure is 0.01 µL cm^{-2} s^{-1}. This very low hydrogen generation rate is consistent with the lack of any observation of bubbles being generated at low heat fluxes despite a voltage of −2 V applied. It could be argued that even a small generation rate can promote nucleation by providing a seed for nucleation. However, no promotion of nucleation in the case of NaBr suggests that electrolysis is not large enough for this to be the case. Tests where voltages of a magnitude greater than 2.0 V also demonstrated a tunable boiling effect; however, bubbles spontaneously formed when $T_{\mathrm{wall}} < T_{\mathrm{sat}}$, indicating appreciable electrolysis affecting nucleation density. Though, using electrolysis to open up nucleation sites may be another useful active method of boiling enhancement with the cost of replenishing lost water and venting generated hydrogen and oxygen.

Supplementary Note 3

Concentration Selection

Surfactant solutions below the critical micelle concentration (CMC) are monomeric (single molecules without any aggregates) while above the CMC, surfactants aggregate into micelles. The CMC is typically a very small concentration on the order of a few mM; therefore, below the CMC many bulk properties such as viscosity, thermal conductivity, specific heat, and saturation temperature are virtually unaffected [6–9]. The CMCs of surfactants used in this study are listed in Supplementary Table 1. Boiling enhancement typically increases up to the CMC [10]; thus, the highest concentration below the lowest CMC surfactant (MEGA-10) with uncertainty was chosen (2.6 mM).

One thermal property that changes with addition of an additive is the boiling point, which can be calculated according to the Clausius-Clapeyron relation and Raoult's Law:

$$\Delta T_{\mathrm{sat}} = K b_{\mathrm{solute}} i \tag{3}$$

Here, K is the ebullioscopic constant (0.512 °C kg mol^{-1} for water), b is the molality of the solute, and i the van 't Hoff factor (2 for completely dissociated diatomic salts). For 2.6 mM concentration of completely dissociated salt, the boiling point elevation is a negligible 2.7×10^{-3} °C. On the other hand, surface energies are altered due to the tendency of surfactants to adsorb at interfaces. Since the concentrations used were all submicellar ($C_1 < C_{\mathrm{CMC}}$), all fluid properties except for surface tension, γ_{lv}, were assumed to be invariant with surfactant concentration.

Supplementary Note 4

Electrohydrodynamic Effects

In any fluid system, conservation of mass and momentum require that

$$\frac{\partial \rho}{\partial t} + \nabla \cdot (\rho \mathbf{v}) = 0 \tag{4}$$

$$\rho \left(\frac{\partial \mathbf{v}}{\partial t} + \mathbf{v} \cdot \nabla \mathbf{v} \right) = -\nabla p + \nabla \cdot \mathbf{T} + \rho \mathbf{g} + \mathbf{f}_{\text{e}} \tag{5}$$

where ρ is the mass density, $\mathbf{v}$ is the velocity, p is the pressure μ is the viscosity, $\mathbf{g}$ is the acceleration due to gravity, and $\mathbf{f}_{\text{e}}$ is the body force due to electric fields. The body force within the fluid due to an electric field, $\mathbf{E}$, has two components: a free charge component, $\mathbf{f}_{\text{e,f}}$, and a dielectric component, $\mathbf{f}_{\text{e,d}}$ [11, 12].

$$\mathbf{f}_{\text{e}} = \underbrace{\rho_{\text{f}} \mathbf{E}}_{\mathbf{f}_{\text{e,f}}} + \underbrace{\left[-\frac{1}{2} |\mathbf{E}|^2 \nabla \varepsilon + \nabla \left[\frac{1}{2} \rho |\mathbf{E}|^2 \left(\frac{\partial \varepsilon}{\partial \rho} \right)_T \right] \right]}_{\mathbf{f}_{\text{e,d}}} \tag{6}$$

Here, the absolute permittivity, $\varepsilon = \varepsilon(\rho, T) = \varepsilon_{\text{r}}(\rho, T)\varepsilon_0$, is assumed to have a real number value since complex permittivity is not important until frequencies are on the order of several GHz [13]. The free charge density, ρ_{f}, is the net difference between positive and negative charge. Since free charges in the conductor must populate at the surface, ρ_{f} is zero everywhere in the bulk. Only in the vicinity of the electric double layer ($\delta_{\text{dl}} \approx 5\,\text{nm}$ in our system) will free charge density play a role. The dielectric term, $\mathbf{f}_{\text{e,d}}$, in equation (6) depends on gradients in density and temperature which only appear in the thermal boundary layer. The thermal boundary length can be estimated using $\delta_{\text{th}} = 3.22k/h_{\text{boil}}$ [14]. At the largest heat transfer coefficients observed in our experiments (approximately $4\,\text{W}\,\text{cm}^{-2}\,\text{K}^{-1}$), the thermal boundary length is calculated to be 0.05 mm. Since $\delta_{\text{th}} >> \delta_{\text{dl}}$, we ignore any contribution due to free charge density. To estimate the effect of electrostriction (dielectric body force effects) in the boundary layer, we convert equation (6) to 1D and apply $\frac{dT}{dx} \approx -\frac{T_{\text{wall}} - T_{\text{sat}}}{\delta_{\text{th}}}$:

$$f_{\text{e,d}} = -\frac{1}{2} |\mathbf{E}|^2 \frac{dT}{dx} \left[\left(\frac{\partial \varepsilon}{\partial T} \right)_\rho + 2 \left(\frac{\partial \varepsilon}{\partial \rho} \right)_T \left(\frac{\partial \rho}{\partial T} \right)_p + \rho \frac{\partial^2 \varepsilon}{\partial \rho \partial T} \right] \tag{7}$$

$$f_{\text{e,d}} \approx \frac{1}{2} |\mathbf{E}|^2 \frac{T_{\text{wall}} - T_{\text{sat}}}{\delta_{\text{th}}} \left[\left(\frac{\partial \varepsilon}{\partial T} \right)_\rho + 2 \left(\frac{\partial \varepsilon}{\partial \rho} \right)_T \left(\frac{\partial \rho}{\partial T} \right)_p + \rho \frac{\partial^2 \varepsilon}{\partial \rho \partial T} \right] \tag{8}$$

Evaluating $f_{\text{e,d}}$ at a saturation temperature of $100\,^\circ\text{C}$ yields a negligible value of $0.5\,\text{mN}\,\text{m}^{-3}$, which is more than six orders of magnitude smaller than the gravitational body force. Thus, EHD effects in the bulk and boundary layer are completely negligible.

Since free charges accumulate at the surface (liquid-vapor and liquid-solid interfaces) at a very small characteristic

Debye length, δ_{dl}, the free charge body force, $f_{\mathrm{e,f}}$, can affect interfacial tension [15]. Mugele shows that incorporating equation (6) into the stress tensor and integrating yields a change in solid-liquid interfacial tension.

$$\gamma_{\mathrm{sl,eff}} = \gamma_{\mathrm{sl}} - \frac{1}{A}\int_0^Q \phi_{\mathrm{d}}(q)\, dq \tag{9}$$

The $\int_0^Q \phi_{\mathrm{d}}(q)\, dq$ term is the energy stored in the electric double layer (equivalent to $CV^2/2$ in a regular capacitor) where ϕ_{d} is the diffuse layer voltage. Using Gouy-Chapman-Stern (GCS) theory, an expression of the diffuse layer voltage can be obtained for a simple $z:z$ electrolyte:

$$\phi_{\mathrm{d}}(\sigma) = \frac{2k_{\mathrm{B}}T}{ze}\sinh^{-1}\left(\frac{\sigma}{2\sqrt{2}\sqrt{c_0 RT\varepsilon\varepsilon_0}}\right) \tag{10}$$

Here, σ is the surface charge density ($\mathrm{A\,m^{-2}}$) and c_0 is the bulk concentration of electrolyte. Using the simple substitution $dq = d\sigma A$, equation (9) can be expressed as

$$\gamma_{\mathrm{sl,eff}} = \gamma_{\mathrm{sl}} - \int_0^\sigma \frac{2k_{\mathrm{B}}T}{ze}\sinh^{-1}\left(\frac{s}{\sqrt{8c_0 RT\varepsilon\varepsilon_0}}\right) ds \tag{11}$$

$$\gamma_{\mathrm{sl,eff}} = \gamma_{\mathrm{sl}} - \frac{2k_{\mathrm{B}}T}{ze}\left[\sqrt{8c_0 RT\varepsilon\varepsilon_0} - \sqrt{8c_0 RT\varepsilon\varepsilon_0 + \sigma^2} + \sigma\sinh^{-1}\left(\frac{\sigma}{\sqrt{8c_0 RT\varepsilon\varepsilon_0}}\right)\right] \tag{12}$$

The integral term is always positive; thus, the effective solid-liquid interfacial energy is always reduced. As a consequence, any electrowetting effect always works to reduce the contact angle of the fluid and increase wetting regardless of the polarity of applied potential. From nucleation theory, any decrease in contact angle should suppress bubble nucleation. However, in the NaBr control experiment, nucleation was not visibly suppressed nor did the boiling curve change substantially. The fact that the contact angle actually increased and nucleation was promoted with DTAB under applied voltage indicates that EHD or electrowetting effects are minimal at the solid-liquid interface. While using GCS theory, it is possible to determine a value of interfacial tension decrease, GCS theory breaks down at higher voltages and during electrolysis. Therefore, the amount of electrowetting cannot be accurately determined, other than observations that show that its effects are minimal.

Supplementary References

1. Sulthana, S. B. *et al.* Solution Properties of Nonionic Surfactants and Their Mixtures: Polyoxyethylene (10) Alkyl Ether [C_nE_{10}] and Mega-10. *Langmuir* **16,** 980–987 (2000).
2. Zoeller, N. J. & Blankschtein, D. Development of user-friendly computer programs to predict solution properties of single and mixed surfactant systems. *Ind. Eng. Chem. Res.* **34,** 4150–4160 (1995).
3. Carey, V. P. *Liquid Vapor Phase Change Phenomena: An Introduction to the Thermophysics of Vaporization and Condensation Processes in Heat Transfer Equipment* 2nd ed. (Taylor & Francis, 2007).
4. Mikic, B. B. & Rohsenow, W. A new correlation of pool-boiling data including the effect of heating surface characteristics. *J. Heat Transfer* **91,** 245 (1969).
5. Lorenz, J. J. *The effects of surface conditions on boiling characteristics* PhD thesis (Massachusetts Institute of Technology, 1972).
6. Cheng, L., Mewes, D. & Luke, A. Boiling phenomena with surfactants and polymeric additives: A state-of-the-art review. *Int. J. Heat Mass Transfer* **50,** 2744–2771 (2007).
7. Hetsroni, G. *et al.* The effect of surfactants on bubble growth, wall thermal patterns and heat transfer in pool boiling. *Int. J. Heat Mass Transfer* **44,** 485–497 (2000).
8. Wang, A. & Hartnett, J. P. Influence of surfactants on pool boiling of aqueous polyacrylamide solutions. *Wärme-und Stoffübertragung* **27,** 245–248 (1992).
9. Musbally, G. M., Perron, G. & Desnoyers, J. E. Apparent molal volumes and heat capacities of ionic surfactants in water at 25C. *J. Colloid Interface Sci.* **48,** 494–501 (1974).
10. Wasekar, V. & Manglik, R. The influence of additive molecular weight and ionic nature on the pool boiling performance of aqueous surfactant solutions. *Int. J. Heat Mass Transfer* **45,** 483–493 (2002).
11. Landau, L. D. & Lifshitz, E. M. *Electrodynamics of Continuous Media* (Pergamon Press, Oxford, New York, 1960).
12. Jones, T. B. Electrohydrodynamically enhanced heat transfer in liquids- A review. *Adv. Heat transfer* **14,** 107–148 (1978).
13. Kaatze, U. Complex permittivity of water as a function of frequency and temperature. *J. Chem. Eng. Data* **34,** 371–374 (1989).
14. Tien, C. L. A hydrodynamic model for nucleate pool boiling. *Int. J. Heat Mass Transfer* **5,** 533–540 (1962).
15. Mugele, F. & Baret, J.-C. Electrowetting: from basics to applications. *J. Phys.: Condens. Matter* **17,** R705–R774 (2005).
